# The cost-effectiveness of penicillin allergy assessment pathway (PAAP): a decision analysis

**DOI:** 10.1136/bmjopen-2025-104168

**Published:** 2025-12-03

**Authors:** Miaoqing Yang, Rebecca Bestwick, Daniel Howdon, S Ahmed, Neil Powell, Kelsey Fiona Armitage, Joanne Fielding, Catherine E Porter, Sinisa Savic, Robert M West, Philip Howard, Ushma Galal, Sue Pavitt, Jonathan AT Sandoe, Ruben Ernesto Mujica-Mota

**Affiliations:** 1 Leeds Institute of Health Sciences, University of Leeds, Leeds, UK; 2 St James’s University Hospital, Leeds, UK; 3 Royal Cornwall Hospitals NHS Trust, Truro, UK; 4 Nuffield Department of Primary Care Health Sciences, Clinical Trials Unit, University of Oxford, Oxford, UK; 5 Dental Translational and Clinical Research Unit, School of Dentistry, University of Leeds, Leeds, UK; 6 Clinical Immunology and Allergy, University of Leeds, Leeds Institute of Medical Research at St James’s, Leeds, UK; 7 School of Health Care, University of Leeds, Leeds, UK; 8 Nuffield Department of Primary Care Health Sciences, University of Oxford, Oxford, UK; 9 Department of Microbiology, University of Leeds, Faculty of Medicine and Health, Leeds, UK; 10 Leeds teaching Hospitals NHS Trust, Leeds, UK; 11 Health and Community Sciences, University of Exeter, Exeter, UK

**Keywords:** HEALTH ECONOMICS, Health Care Costs, Quality of Life, Primary Care

## Abstract

**Objective:**

To evaluate the cost-effectiveness of implementing a penicillin allergy assessment pathway (PAAP) versus usual care within the NHS.

**Design:**

A decision tree analysis over a 5-year time-period, informed by a randomised controlled trial (RCT) of PAAP and systematic review. Value of information analysis was also conducted to estimate the value of conducting a new trial.

**Data sources:**

Model inputs were informed by the ALABAMA RCT participants included in the primary analysis, 811 adults with penicillin allergy labels and recent antibiotic prescriptions, and data from published literature.

**Interventions:**

Participants in the ALABAMA trial included in the primary analysis: PAAP (n=401) and usual care (n=410).

**Primary and secondary outcome measures:**

Costs are presented in GBP (£) at 2022–2023 prices, quality-adjusted life years (QALYs), incremental cost-effectiveness ratio, incremental net monetary benefit (INMB), the probability of cost-effectiveness at the £20,000 and £30,000 per QALY threshold, and the cost effectiveness of a new follow-on trial.

**Results:**

PAAP had incremental costs of £−83 (probability of cost saving 47.5%) and incremental QALYs of 0.036 (probability of positive benefits 47.5%). The INMBs (probability of cost-effectiveness) were £806 (48%) and £1167 (48%) under the decision thresholds of £20,000 and £30,000 per QALY, respectively. PAAP was more cost-effective among females, people aged >65 years, and more frequent antibiotic users. A new follow-on trial involving 1267 participants was estimated to cost £2.4 million and, by reducing uncertainty in the evidence, would avoid £19.6 million in costs of incorrect management decisions for eligible patients over the next 10 years.

**Conclusion:**

The PAAP was considered cost-effective, but significant uncertainty remained. Future trials with adequate power and longer follow-up are needed to determine the most cost-effective models for penicillin allergy testing.

**Trial registration number:**

ISRCTN20579216.

**STRENGTHS AND LIMITATIONS OF THIS STUDY**

- First economic evaluation of penicillin allergy testing based on high-quality randomised controlled trial.
- Relatively short modelling time horizon of 5 years.
- Relatively low power to show the cost-effectiveness of penicillin allergy assessment pathway among subgroups.

## Introduction

 Penicillins, the most prescribed antibiotics, are first-line therapy for many infections. However, recorded penicillin allergies significantly influence antibiotic selection. Approximately 6% of the UK general practice population has recorded penicillin allergies, often incorrect due to mis-labelling infection-related symptoms as allergies.^1^ Penicillin allergy records in primary care patients are associated with suboptimal antibiotic therapy, increased antimicrobial resistance and poorer health outcomes.^2^

Implementing a pre-emptive penicillin allergy assessment pathway (PAAP) initiated in primary care could improve patient care, reduce healthcare-associated infections and generate NHS cost savings. A PAAP intervention, if widely implemented, could increase the number of people assessed and shift assessment from specialists to delivery by a wider group of healthcare professionals. While there are potential health and cost implications for patients who test negative but later react to penicillin, evidence suggests these risks are small.^3^ There are also potential savings from using penicillins instead of broader-spectrum antibiotics and reduced primary and secondary care use.^4^

No previous study has investigated the long-term cost-effectiveness of penicillin allergy assessment based on evidence from a randomised controlled trial (RCT). Building on our previously published within-trial analysis of the ALABAMA RCT, this study presents a modelled 5-year economic evaluation of the PAAP, assessing its impact on healthcare costs, patient health-related quality of life (HRQoL), and long-term cost-effectiveness in patients with a record of penicillin allergy.^5 6^ The original primary objective of the ALABAMA trial was to assess whether PAAP is clinically effective in reducing treatment response failure, but early termination of recruitment due to COVID-19 prevented attainment of the target sample to test this outcome. We have separately reported the within-trial cost-effectiveness analysis of PAAP at 12 months and herein present cost-effectiveness analysis of PAAP extended over 5 years and value of information analysis.^6^

## Methods

We developed an economic model to assess the cost-effectiveness of implementing PAAP initiated in primary care from the English NHS perspective over a 5-year time horizon, the maximum number of years for which we had at least partial data on any trial participant (4.3 years) and found resource use data in our systematic review of economic studies of penicillin allergy de-labelling (currently under review for publication).^7^ The modelled population included adults with a recorded penicillin allergy.^5^ A health economic analysis plan was developed and is available on request. The model structure was informed by our systematic review, with model inputs derived from the ALABAMA trial. The statistical analysis methods applied to the trial data, including handling of missing data and subgroup analyses, have been previously described in detail in our published within-trial analysis.^6^ All analyses were conducted using R (V.4.5.1).^8^ The decision tree model was developed following the open-source Decision Analysis in R for Technologies in Health (DARTH) framework for decision-analytic modelling in R (https://github.com/DARTH-git).

### Data sources

We used individual, patient-level data from the ALABAMA trial, including linked primary care electronic health records capturing general practitioner (GP) and nurse consultations and antibiotic prescriptions; Hospital Episode Statistics data covering inpatient admissions, outpatient visits and emergency department attendances; and Office for National Statistics data on registered deaths.

### Costs and health outcomes

The analysis included costs of antibiotic medications, primary care consultations prescribed in primary care, inpatient hospital admissions, including episodes spent in critical care, accident and emergency visits and outpatient attendances. Costing of the PAAP intervention incorporated data on staff time valued using NHS staff salaries, with tests, consumables and other materials valued at prices paid by Leeds Teaching Hospitals NHS Trust, the main study site during the trial (2023–2024). Costs are presented in GBP (£) at 2022–2023 prices. Health outcomes were measured using EQ-5D-5L responses at baseline and 12 months, and at 3 days and 28 days after the start of any (selected) antibiotic medication.^5^ Responses were mapped to EQ-5D-3L utility values.^9^

Costs and quality-adjusted life years (QALYs) were discounted at an annual rate of 3.5%. Results were presented in terms of the incremental cost-effectiveness ratio (ICER), incremental net monetary benefit (INMB) and the probability of cost-effectiveness at the £20,000 and £30,000 per QALY thresholds.

### Model structure and data inputs

**Figure 1:**
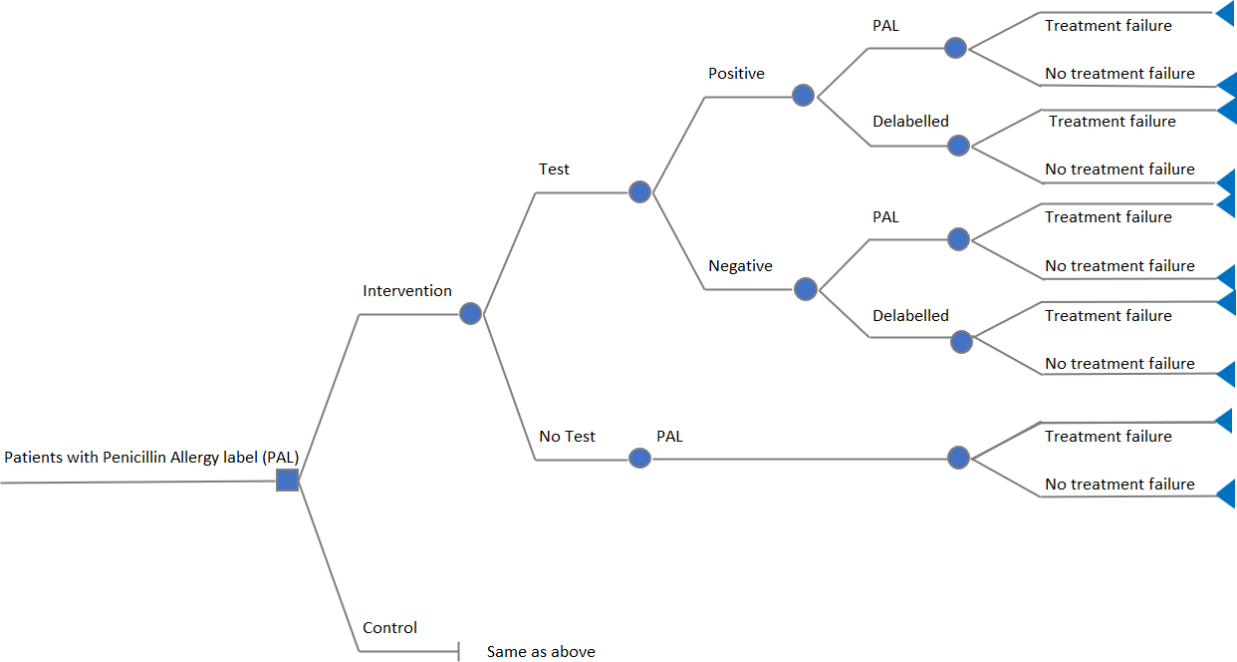
Structure of the decision tree model. Test result interpretation: a ‘Positive’ result is a test which indicates patient is penicillin allergic. ‘Negative’ result is a test which indicates a patient is not allergic. PAL, Penicillin Allergy Label.

**Figure 2:**
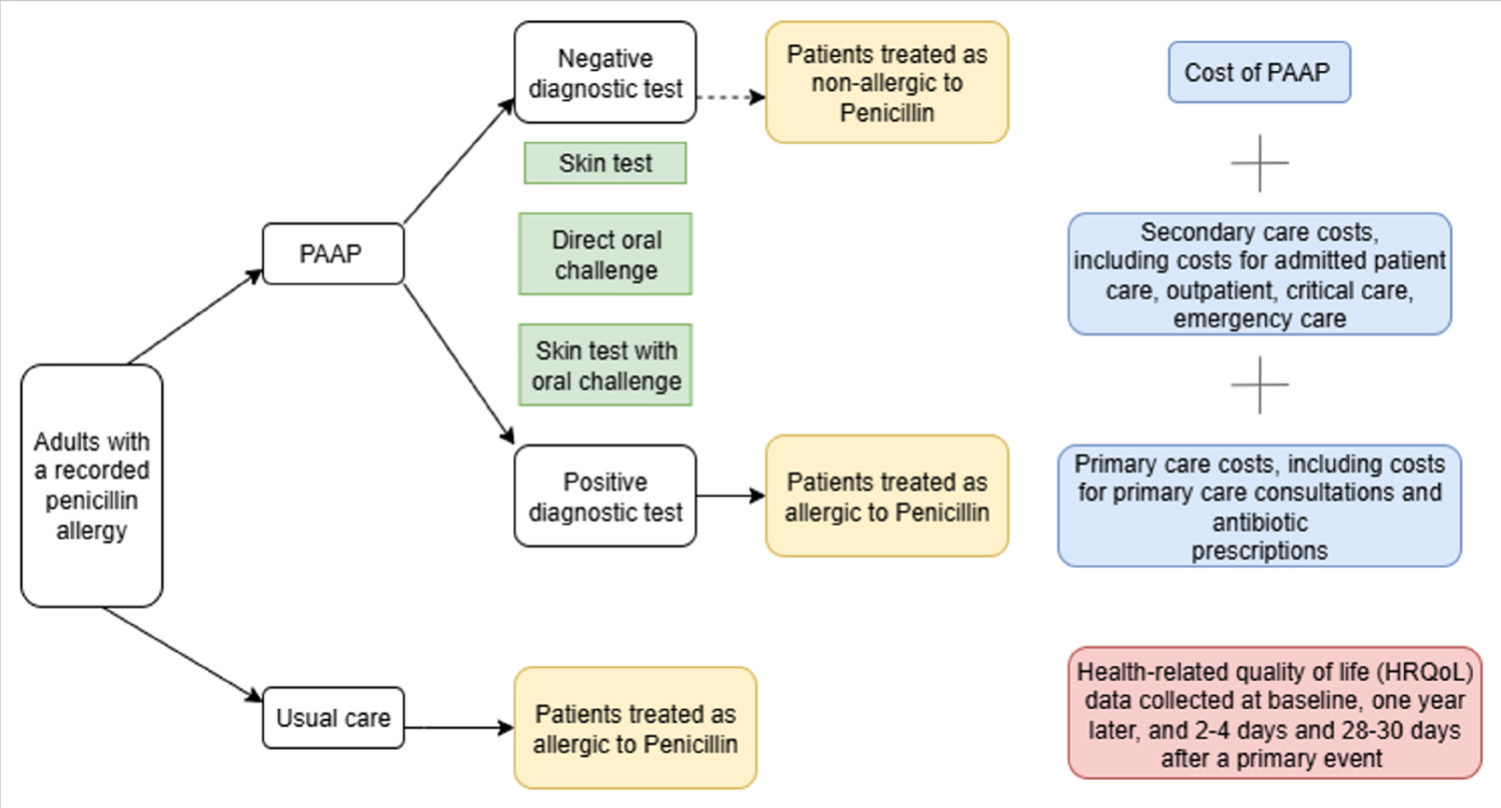
Graphical description of the pathways and data for modelling. PAAP, penicillin allergy assessment pathway.

We built a decision tree model to extrapolate trial results beyond the end of the 12-month trial to 5 years. Figure 1 presents the structure of the decision tree model, and figure 2 illustrates the treatment pathway and identifies the data sources used to inform model inputs and assumptions. Most participants (91%) in the PAAP arm of ALABAMA underwent penicillin allergy testing (hereafter referred to as ‘testing’) whereas only 1.2% did so in the control arm. Most of those who were tested were found not to be penicillin allergic and had their allergy label removed. The model was divided into two parts. In the first part, we modelled the 12-month costs and QALYs of each trial arm according to the testing status (test vs no test), the results of the test (positive vs negative), whether the trial participant had their allergy label removed from the primary care record (de-labelled vs not de-labelled) and whether the patient experienced treatment failure in the first year of ALABAMA. In the second part of the model, we extrapolated costs from 12 months to 5 years using the predicted mean annual costs derived from regression equations fitted to post-12-month individual participant primary and secondary care use and average cost data in ALABAMA, as a function of 12-month penicillin allergy label and treatment failure status. As no post-12-month HRQoL data was collected in ALABAMA nor available from published studies, we assumed QALYs would remain constant at their 12-month values over the 5-year modelled period. The model included data from a cohort of 811 participants: 401 in the treatment group and 410 in the control group (online supplemental table A1.1). The breakdown of observed costs and QALYs used to derive model parameters is presented in table 1.

**Table 1:** Summary resource use and cost data for analysis

Model parameter	PAAP		Usual care	
	Mean	SD	Point estimate	SD
Quantities of service use				
First year	n=401		n=410	
SPT only	0.01	0.09	0.00	0.05
STOC	0.32	0.47	0.01	0.07
DPC	0.58	0.49	0.00	0.05
Primary care consultations	1.21	2.38	1.48	2.68
Primary care medications	1.12	2.10	1.52	2.90
Hospital admissions	0.42	1.11	0.37	0.90
Outpatient visits	2.86	4.87	2.92	5.06
A&E	0.30	0.95	0.35	0.79
Second year	274		278	
Primary care consultations	1.24	2.41	1.32	2.59
Primary care medications	1.28	2.52	1.42	2.94
Hospital admissions	0.37	1.06	0.50	2.68
Outpatient visits	3.42	5.93	3.02	5.47
A&E	0.25	0.71	0.34	1.21
Cost				
First year	n=401		n=410	
Test	169.52	66.31	1.52	16.29
Primary care consultations	38.74	76.23	47.25	85.50
Primary care medications	6.07	29.05	8.87	37.00
Hospital admissions	643.54	2003.29	748.12	2761.80
Outpatient visits	462.59	782.71	475.51	803.88
A&E	74.25	234.29	89.72	223.45
Total	1394.71	2632.52	1370.99	3218.43
Second year*	274		278	
Primary care consultations	39.59	76.77	42.02	82.64
Primary care medications	8.30	39.00	7.29	25.23
Hospital admissions	848.79	3051.78	888.58	5133.87
Outpatient visits	581.54	1051.23	507.67	911.62
A&E	59.11	182.94	73.18	251.95

Observed values in ALABAMA trial multiplied by 365 and divided by the exposure time in days during the annual observation period, to adjust for censoring.

* Data for third, fourth and fifth years were available for a minority of patients and included in the analysis of beyond year 1 annual cost pay-offs (online supplemental file 1).

DPC, direct oral penicillin challenge; PAAP, Penicillin Allergy Assessment Pathway; SD, Standard Deviation; SPT, skin prick test; STOC, skin prick test with oral penicillin challenge.

The methodology for deriving input parameters, including probabilities, costs and QALYs, is detailed in online supplemental file 1. Base-case values are presented in table 2 and online supplemental table A1.4.

**Table 2:** Model parameter values and probability distributions

Model parameter	PAAP (N=401)		Usual care (N=410)		
	Point estimate	SD	Point estimate	SD	Distribution
Event probabilities					
Receive test	0.910	0.082	0.012	0.012	Beta
Test positive	0.082	0.075	0.200	0.160	Beta
PAL removed after positive test	0.000	Fixed	0.000	Fixed	Beta
PAL removed after negative test	0.973	0.026	1.000	Fixed	Beta
TF after test positive and PAL not removed	0.033	0.032	0.000	0.000	Beta
TF after test negative and PAL not removed	0.111	0.099	0.000	0.000	Beta
TF after test negative and PAL removed	0.089	0.081	0.000	0.000	Beta
TF with no test	0.083	0.076	0.114	0.101	Beta
Cost					
First year costs					
Test+, PAL and TF	510.23	0.000	0.00	0.00	Lognormal
Test+, PAL and no TF	1176.55	1587.10	839.63	0.00	Lognormal
Test−, PAL and TF	3565.61	0.00	0.00	0.00	Lognormal
Test−, PAL and no TF	501.37	399.85	0.00	0.00	Lognormal
Test−, no PAL and TF	1868.20	2249.55	0.00	0.00	Lognormal
Test−, no PAL and no TF	1481.03	2694.72	409.14	724.91	Lognormal
No test and TF	432.47	333.95	2077.52	2760.80	Lognormal
No test and no TF	604.89	1063.40	1208.45	3038.71	Lognormal
Annual QALYs					
Test+, PAL and TF	0.924	0.000	0.000	0.000	Beta
Test+, PAL and no TF	0.828	0.226	0.980	0.000	Beta
Test−, PAL and TF	0.776	0.000	0.000	0.000	Beta
Test−, PAL and no TF	0.837	0.132	0.000	0.000	Beta
Test−, no PAL and TF	0.808	0.146	0.000	0.000	Beta
Test−, no PAL and no TF	0.880	0.137	0.888	0.091	Beta
No test and TF	0.689	0.060	0.755	0.249	Beta
No test and no TF	0.902	0.082	0.850	0.175	Beta

These values were used to substitute missing QALY when there was no individual for certain decision tree branches.

PAAP, Penicillin Allergy Assessment Pathway; PAL, penicillin allergy label; QALY, quality adjusted life year; SD, Standard Deviation; TF, treatment failure.

### Deterministic and probabilistic sensitivity analyses

In sensitivity analyses we considered alternative unit costs for PAAP, using the NHS National Reference Costs unit cost for a clinical immunology and allergy day case visit (£368), to approximate costs under implementation of the PAAP as a single day case visit without the need for separate initial clinic review. In separate analysis, we used instead micro-cost estimates from the Leeds Teaching Hospitals NHS Trust Immunology Centre, as Leeds was the primary trial site. We present an ‘As Treated’ analysis, where patients in the PAAP arm who did not undergo testing were re-assigned to the control group. We analyse the scenario where patients are relabelled during years 2–5 at a constant rate in a Markov chain and estimate the annual relabelling rate threshold values for cost saving and for positive net monetary benefit. In addition, we explored a pessimistic scenario where the 6-month relabelling rate of 6.6% observed in the PALACE study remained constant over the 5-year follow-up in the Markov chain model.^10^

All model parameters defined were subject to probabilistic analysis, where the relevant distributions were estimated from the individual-level trial data (online supplemental tables 2 and A1.4). We present the cost-effectiveness plane to depict the uncertainty in the results, along with the cost-effectiveness acceptability curve (CEAC) illustrating the probability of cost-effectiveness as a function of the value of a QALY to policymakers.^11^

### Subgroup analyses

Subgroup analyses were conducted by age (<65 years vs ≥65 years), gender (females vs males), number of quality and outcomes framework (QOF) conditions (<2 vs ≥2) and number of antibiotic prescriptions at baseline (<2 vs ≥2).

### Value of information

The original primary outcome of ALABAMA was treatment response failure (ie, the re-prescription rate of an alternative antimicrobial within 28 days of an index prescription). To detect a difference in this outcome, more participants are needed than were recruited. We therefore assessed the expected value of sample information from a follow-on trial recruiting the required remaining number of trial participants. We compared the costs of the hypothetical new trial with the benefits the new information would generate in reducing the risks of making the wrong decision with the information from ALABAMA alone (online supplemental file 3). We also present the expected value of perfect model parameter information (reducing all parameter uncertainty), to indicate outcomes where research may be most valuable.

### Patient and public involvement

No patients were involved in the planning and development of this study.

## Results

### Base-case analysis

The costs and QALYs for the PAAP intervention compared with usual care are summarised in table 3. In year 1, PAAP incurred slightly higher costs compared with usual care, whereas it saved costs over years 2–5. In terms of health outcomes, PAAP yielded slightly more QALYs than usual care for both year 1 and year 2+.

**Table 3:** Total costs and QALYs per year

	PAAP	Usual care	Difference	95% CI	
				Lower	Upper
Costs year 1 (£)	684.141	630.854	53.287	11.529	95.044
Costs year 2–5 (£; discounted)	1523.834	1660.246	−136.412	−142.982	−129.843
QALYs year 1	0.429	0.422	0.008	0.005	0.011
QALYs year 2–5 (discounted)	1.577	1.549	0.028	0.018	0.039

CI, Confidence Interval; PAAP, Penicillin Allergy Assessment Pathway; QALYs, quality-adjusted life years.

PAAP had lower total healthcare expenditure compared with usual care, indicating that the savings from removing incorrect penicillin labels outweighed the higher intervention costs over a 5-year horizon (table 4). PAAP also had higher QALYs as more patients were tested and de-labelled than usual care. This resulted in the PAAP being a dominant strategy over usual care. At willingness-to-pay thresholds of £20,000 and £30,000 per QALY, the INMB was £564 and £788 per patient, respectively.

**Table 4:** Summary results of probabilistic sensitivity analysis

	Incremental costs (£)	Incremental QALYs	ICER (£/QALY)	INMB (probability of being cost-effective)	
				£20 000	£30 000
Base case (n=811)	−114.959	0.022	Dominant	563.834	788.272
PSA (n=811)	−83.125	0.036	Dominant	805.723 (48%)	1167.022 (48%)

ICER, incremental cost-effectiveness ratio; INMB, incremental net monetary benefit; PSA, probabilistic sensitivity analysis; QALY, quality-adjusted life year.

### Probabilistic sensitivity analysis

In the probabilistic sensitivity analysis, PAAP had a slightly smaller costing saving of £83 but a larger QALY gain of 0.036 than under the base-case analysis. The INMB values were £806 at £20,000 and £1167 at £30,000 per QALY decision thresholds. The probability of the PAAP intervention being cost-effective was 48% at the £20,000 and the £30,000 per QALY thresholds (see cost-effectiveness plane and CEAC in online supplemental file 4).

### Sensitivity analyses

When alternative cost assumptions were applied, such as HRG tariff costing and Leeds-specific PAAP delivery, the PAAP intervention still yielded positive INMBs (table 5). As before, the probabilities of being cost-effective were all slightly below 50%. In contrast, the ‘As Treated’ analysis produced QALY and net benefit losses, and probabilities of cost-effectiveness as low as 20%. Among the 36 patients in the intervention group who did not receive tests, QALYs were higher than the average, which led to larger QALYs in the control group when these patients were reassigned to the control group. In the scenario with relabelling, higher re-labelling rates are associated with lower PAAP cost-effectiveness; the probability that the largest relabelling rate reported in the literature (3.2% Macy *et al*^12^; Du Plessis *et al*^13^)is below the rate at which the PAAP INMB becomes negative is 82%; the respective figure for PAAP becoming cost increasing is 59%. When the Markov chain model is revised to allow for 6.6% 6-month relabelling rate that remains constant over 5 years, the (deterministic) ICER is equal to £1122 per QALY gained.

**Table 5:** Sensitivity analyses

	Incremental costs (GBP)	Incremental QALYs	ICER (£/QALY)	INMB (probability of being cost-effective)	
				£20,000	£30,000
HES costing (n=811)	16.322	0.036	451.755	706.276 (48%)	1067.575 (48%)
‘As Treated’ (n=811)	−435.792	−0.350	1243.559*	−6572.997 (20%)	−10077.390 (19%)
HES costing among the ‘As Treated’ (n=811)	−370.078	−0.350	1056.041*	−6638.711 (20%)	−10143.110 (19%)
Leeds PAAP delivery (n=811)	−84.781	0.036	Dominant	807.379 (48%)	1168.678 *(*48%)

* The interpretation of this cost-effectiveness ratio is different from the standard case because the intervention saves costs and reduces QALYs, that is, the amount of saving per QALY lost by the intervention.

ICER, incremental cost-effectiveness ratio; INMB, incremental net monetary benefit; QALY, quality-adjusted life year.

### Subgroup analysis

PAAP is more cost-effective for certain groups, particularly females, older adults, individuals with fewer comorbidities (QOF registered conditions) and frequent antibiotic users. For these subgroups, the probability of PAAP being cost-effective exceeds 50% (table 6). Notably, among females and those aged 65+ years, PAAP saved costs and generated more QALYs than usual care. However, for males, younger patients, those with more comorbidities and less frequent antibiotic users, PAAP has a lower probability of being cost-effective, below 50%.

**Table 6:** Subgroup analyses

	Incremental costs (£)	Incremental QALYs	ICER (£/QALY)	INMB (probability of being cost-effective)	
				£20,000	£30,000
Male (n=227)	−264.00	−0.018	14893.580*	−90.514 (45%)	−267.771 (44%)
Female (n=584)	−20.051	0.047	Dominant	967.089 (52%)	1440.607 (52%)
Age <65 years (n=555)	−79.243	−0.005	15123.050^*^	−25.555 (44%)	−77.953 (44%)
Age ≥65 years (n=256)	−60.956	0.120	Dominant	2455.833 *(*58%)	3653.272 *(*58*%*)
QOF <2 (n=403)	117.570	0.262	448.397	5126.438 (77%)	7748.442 (78%)
QOF 2+ (n=408)	−335.584	−0.198	1694.506*	−3625.266 (32%)	−5605.690 (31%)
Abx <2 (n=462)	−220.796	−0.111	1982.562*	−2006.583 (36%)	−3120.273 (36%)
Abx 2+ (n=349)	37.615	0.229	164.169	4544.862 (68%)	6836.101 (68%)

* Unlike the standard ICER situation of its producing higher QALYs and higher costs, the intervention produces fewer QALYs and lower costs and its ICER is therefore interpreted as the cost saving per QALY lost in this subgroup relative to usual care.

Abx, antibiotics; ICER, incremental cost-effectiveness ratio; INMB, incremental net monetary benefit.; QALY, quality-adjusted life year; QOF, quality and outcomes framework.

### Value of information analyses

**Figure 3:**
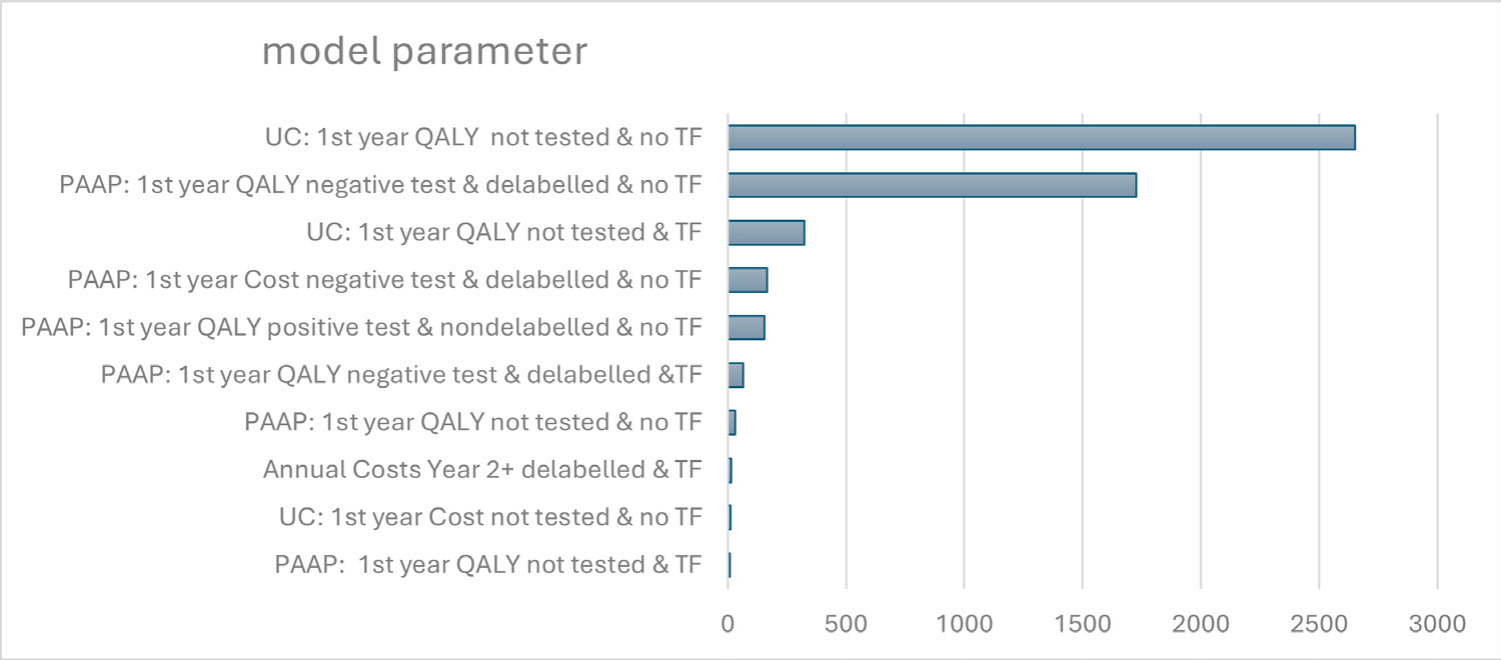
Top 10 outcomes for the expected value of further research. Estimated using the expected value of perfect parameter information, denoting the value of eliminating uncertainty in individual model parameters estimates through further research. PAAP, penicillin allergy assessment pathway (arm); QALY, quality-adjusted life year; TF, treatment failure (during the first 12 months of study); UC, usual care (arm).

HRQoL outcomes dominated all other model parameters as the most valuable research priority (figure 3). A follow-on RCT to enrol the additional 1267 participants required to achieve the original ALABAMA target sample size to detect a difference in treatment failure with 90% power was estimated to cost £2.37 million and require 46 months to complete. The expected value of sample information on clinical outcomes alone for this new trial is £249.15 per patient and £3097 when costs and HRQoL data are also collected, which at the estimated eligible prevalent and incident population over the next 10 years (n=79,877) results in a net expected (discounted) value of sampling of £17.2 million and £241.5 million, respectively. A trial recruiting the 769 participants to achieve 80% power would cost £1.76 million and take 30 months, and have similar net expected value of sampling. The maximum return would be achieved with a study of 840 participants, at a cost of £1.85 million and net value of sampling of £241.7 million.

## Discussion

Our modelling results suggest that PAAP is a cost-effective strategy compared with usual care. Over a 5-year horizon, the PAAP intervention was associated with both lower healthcare costs and improved quality of life outcomes, making it a dominant strategy and likely cost-effective over usual care. However, results were uncertain, and its probabilities of cost-effectiveness were slightly below 50%. This considerable level of uncertainty, stemming from variability in input parameters estimates, was due to the small sample size, limited follow-up time and the relatively small incremental QALY gain observed with the intervention.

Sensitivity analyses of variations in PAAP delivery costs showed similar results as the base-case analyses, with PAAP having positive incremental net benefit. The only exception occurred when we used day care HRG tariff for penicillin allergy testing to evaluate the costs of PAAP and when patients randomised to the PAAP arm who did not receive testing were re-assigned to the control group for (‘As Treated’) analysis. The group of 36 patients allocated to PAAP who did not receive testing were younger than (mean age 54 vs 51 years), but similar in terms of gender (72% vs 71% female) and total number of courses of antibiotics in the 24 months before baseline (mean 1.92 vs 1.91) to, the ITT PAAP group; 4 (11%) did not want to undergo testing, one attended the outpatient appointment for testing but could not go ahead due to eczema/dermatitis, 17 did not attend the appointment (47%) and the rest (n=14, 39%) had no reason recorded). Unlike the unit costs of PAAP estimated by micro-costing (£234 for skin prick test+oral challenge or £155 for direct oral challenge) used in the base-case analysis, the day case HRG tariff cost (£368) is not specific to penicillin allergy testing and type of staff performing the tests. The ‘As Treated’ analysis results surprisingly suggest that, on average, patients on the PAAP arm who were not tested may have had a better prognosis than patients allocated to PAAP who did follow through with their allocated treatment as intended. However, the reallocation in the ‘As Treated’ analysis may have disrupted the original randomisation process and introduced imbalance between groups.

PAAP was particularly cost-effective among females, the elderly and frequent antibiotic users. In these subgroups, the intervention consistently resulted in cost savings and more QALYs than usual care. These results suggest targeting the PAAP model to, say, all males over 65 years and females older than 40 years who have used antibiotics in the past 12–24 months may yield the greatest per-patient net health benefit at a population level with existing health system resources and relieve pressure on overstretched allergy testing services.^14 15^

Ours is the first economic evaluation of penicillin allergy testing based on RCT effectiveness data. A previous observational study compared the healthcare service use of Kaiser Permanente members over 3.6-year follow-up period after receiving penicillin allergy testing with matched controls and found the cases to have 0.09 fewer outpatient department visits and 0.13 fewer emergency department visits per year.^4^ The respective estimates of 0.06 and 0.04 fewer such visits in the within-trial analysis that underpins our results corroborate the direction of the observational effects, although they are significantly smaller. Like ours, a previous study investigated the cost-effectiveness of penicillin allergy testing in a European outpatient setting over a 5-year time horizon using a decision model.^7^ Contrary to our results, it found that penicillin allergy testing was cost-saving, based on an observed 2.2 extra GP contacts per year (median 5.5 vs 3.3) among 1254 penicillin allergic patients relative to a group of 3756 non-penicillin allergic controls in electronic medical records from GP practices in Utrecht, The Netherlands. However, our data do not support the inference that de-labelling patients would reduce this amount of primary care service use. Within the trial, 12-month results from ALABAMA found the penicillin allergy test group to have a mean of 1.20 consultations versus 1.45 in the usual care group^6^; in our analysis for the extrapolation model using post 12 months trial data, we found the predicted annual mean number of consultations to be 1.33 and 1.36, respectively, for patients who had and had not been de-labelled by the 12-month trial end point.

Our analysis uses a decision tree to model observed outcomes in the first year of ALABAMA and extrapolation to derive outcomes for the second to the fifth year following randomisation. The model is therefore unable to account for the potentially life-long benefits of improved access to first-line treatment after de-labelling and more likely to underestimate benefits among younger population subgroups. Furthermore, the small trial sample prevented us from investigating the intersections of subgroup characteristics, such as older age and comorbidity and higher use of antibiotics, to identify groups for more targeted intervention. Our number of QOF-recorded conditions in primary care measure included blood pressure tests performed for around two-thirds of the sample, which may have contributed to the unexpected finding of a lower cost-effectiveness likelihood of PAAP among those with more than two conditions than other patients and warrants further research with appropriate measures of chronic condition burden. In extrapolating costs and QALYs, our model relied on the assumption that healthcare service costs are driven by treatment failure during the 12-month trial period (the primary outcome in the original trial design) and penicillin allergy label status at the end of it. In our trial participant-linked primary and secondary care data for the second and subsequent years, participants who had no label at 12 months had 6%–11% lower annual costs than participants with a label. Furthermore, those with recorded treatment failure in the 12 months after randomisation had 39%–46% higher annual costs over the following 2–3 years than other trial participants. These estimates drove the longer-term results in our model and captured impacts on medication use, primary care visits, inpatient admissions and critical care, emergency care visits and outpatient secondary care, thus integrating outcomes whose association with penicillin allergy labels has been the focus of extensive but fragmented attention in the literature.^16^

Contrary to previous studies reporting the occurrence of relabelling in 1.6%^13^ and 3.2%,^12^ there were no such cases in ALABAMA, which may be an artifice of the controlled setting of the study. Allowing for the relabelling at previously reported rates would not alter the conclusions that PAAP is expected to save costs and be cost-effective.

Our study is based on data from the ALABAMA RCT, which halted participant recruitment early due to challenges brought by COVID-19. The premature termination of ALABAMA is reflected in the wide CIs around our estimates of PAAP effects on secondary care costs and probability of cost-effectiveness, which lies around 50%. The trial took place during a period of significant disruption to healthcare delivery, with many patients avoiding GP contact and antibiotic prescribing rates falling. These changes likely reduced the number of primary events and may have led to an underestimation of the true effect of intervention. Moreover, HRQoL outcomes were collected at baseline and trial end, which for many participants were less than 12 months, limiting the robustness of long-term economic modelling. Extrapolating healthcare use data from this pandemic period to future years is inherently uncertain, and the generalisability of findings to post-pandemic settings is affected. Our study suggests that the value of conducting a new trial involving the 1297 planned participants that remained to be recruited after trial termination is 10 times its cost, while 840 participants would have maximum value and cost £1.85 million.

Other service delivery models, including through the use of decision support tools, such as ‘PEN-FAST’ in the PALACE trial,^10^ may impact on cost effectiveness, but this would need prospective study. Primary care delivered de-labelling may further increase the cost-effectiveness by avoiding the need to travel to hospital, but this would also need prospective evaluation.

## Conclusion

PAAP intervention is likely to be cost-effective, consistently producing both cost savings and health benefits. However, further research with larger sample sizes and longer follow-up may be necessary to reduce the uncertainty in our analyses and confirm its cost-effectiveness.

### Highlights box

- Recorded penicillin allergies are common and often incorrect, leading to less effective treatments and contributing to antimicrobial resistance.
- This study shows that the PAAP is likely to be cost-effective over 5 years, though uncertainty remained.
- The analysis suggests that adequately powered trials with longer follow-up are needed and would cost less than the estimated £19.6 million in healthcare and quality of life losses associated with current suboptimal management over 10 years.
- The findings support cautious but evidence-informed consideration of PAAP implementation, emphasising the importance of accounting for cost-effectiveness uncertainty in future policy decisions.

## Supplementary material

10.1136/bmjopen-2025-104168 online supplemental file 1

## Data Availability

Data are available upon reasonable request.
