# Supplementary material for: The cost-effectiveness of penicillin allergy assessment pathway (PAAP): a decision analysis
Source: BMJ Open. 2025 Dec 3;15(12):e104168. doi: 10.1136/bmjopen-2025-104168 (PMC12682163; doi:10.1136/bmjopen-2025-104168)

**Supplementary File 1. Model Parameter Values**

#### Baseline characteristics

Baseline characteristics of the sample are shown in Table A1.1 between the usual care and PAAP groups across both the intention-to-treat (ITT) and as-treated populations.

**Table A1.1: Baseline characteristics for intention to treat and as treated analyses**

|  | **ITT** | | **As treated** | |
| --- | --- | --- | --- | --- |
|  | **Usual care n=401** | **PAAP**  **n=410** | **Usual care n=446** | **PAAP**  **n=365** |
| **Variables** | **Mean**  **(SD)** | **Mean**  **(SD)** | **Mean**  **(SD)** | **Mean**  **(SD)** |
| **Age** | 54.68  (15.31) | 54.35  (15.92) | 54.28  (15.42) | 54.81  (15.85) |
| **Females (%)** | 292 (71) | 292 (73) | 318 (71.3) | 266 (72.9) |
| **Number of QOF conditions** | 1.78  (1.40) | 1.73  (1.42) | 1.76  (1.38) | 1.75  (1.44) |
| **Number of antibiotics** | 1.84  (1.65) | 1.91  (1.51) | 1.85  (1.64) | 1.91  (1.51) |

*Notes:* SD: Standard Deviation; ITT: Intention-to-treat.

#### Probabilities

Probabilities for the decision tree model were derived from individual participant data collected during the trial from baseline to the 12-month follow-up. In the treatment group, 363 participants received tests (skin test and/or oral challenge test) as part of the PAAP intervention, while five participants were tested in the usual care arm. We then calculated conditional probabilities for each group based on the test results, whether they were de-labelled and whether they experienced treatment failure. Note that no individuals were de-labelled with positive test results; hence, the probabilities for the associated decision tree branches were set as zero.

There were four patients who were relabelled after de-labelling. Two of these were relabelled by mistake due to a clinic letter sent to the GP containing outdated allergy status; both were de-labelled again shortly afterwards and thus we still categorised them as de-labelled patients. Another patient was initially de-labelled because they reported no reaction on day 5 post-testing, so the test was marked negative and the label was removed per protocol. However, they had a reaction the following day and were subsequently relabelled; this patient was treated as a non-de-labelled patient. The final patient was relabelled more than two years after de-labelling, near the end of the trial. Since the impact of this relabelling was not sufficiently captured by the trial data, we categorised the patient as a de-labelled individual.

#### Intervention cost

The costs of the PAAP intervention were calculated using retrospective data on staff time at Leeds and Truro allergy testing clinics, covering training, screening, risk stratification, and testing via skin test and/or oral challenge. Medications and consumables were estimated based on standard operating procedures aligned with routine practice at Leeds Teaching Hospital Trust. The staff time data were validated using prospective data from Leeds and Bradford clinics, including detailed time records for various testing procedures. Staff time was valued according to Title and Grade using 2022-2023 PSSRU unit costs and 2023 Agenda for Change salaries^11^. Antibiotic costs were based on hospital acquisition prices from the eMIT database (February 2024). Due to variations in staff skill mix across the four testing centres, costs were adjusted accordingly. Testing costs for high and low-risk patients are detailed by study centre in Table A1.2.

**Table A1.2: Unit costs of PAAP by test in study allergy centres**

| Test | Allergy centre | | | |
| --- | --- | --- | --- | --- |
|  | Leeds | Sheffield | Bradford | Truro |
| SPT + OC | 234 | 218 | 272 | 324 |
| SPT | 137 | 129 | 158 | 177 |
| OC | 155 | 145 | 179 | 210 |

*Note:* Estimated from antibiotic medications, consumables and staff time inputs into PAAP delivery at the Leeds allergy centre, valued at staff time cost per hour according to the Title and Grade of staff delivering the intervention at each centre. SPT + OC: Skin Prick test plus oral challenge. OC: Oral challenge test.

#### Costs and QALYs for year 1

Costs for year 1 were calculated using trial data linked with administrative sources, mainly Hospital Episode Statistics (HES) for secondary care and OpenClinica and SystmOne for primary care service and prescribed antibiotic use. The secondary care costs included costs for admitted patient care, outpatient, critical care, emergency care, while primary care costs included costs for primary care consultations and antibiotic prescriptions. Due to the early termination of the ALABAMA trial, some patients were followed for fewer than 365 days. To address incomplete follow-up data, we partitioned the first year cost and QALY data into monthly “buckets” and applied Seemingly Unrelated Regression (SUR) for each month separately, adjusting for treatment allocation, age, number of antibiotic prescriptions in the 24 months prior to randomisation, number of Quality and Outcomes Framework registered diseases and GP practice dummies^12^. All covariates were consolidated into four indices using principal component analysis, transforming categorical variables into continuous ones to facilitate the calculation of the variance-covariance matrix of estimated coefficients in SUR. We then estimated the monthly cost using partitioned regression coefficient estimates for each month and summed the predicted costs across 12 months to obtain the first-year cost.

Utility measures for year 1 were based on health-related quality of life (HRQoL) data collected from trial participants at baseline, one year later, and 2-4 days and 28-30 days after a primary event, in which an antibiotic was prescribed for a pre-defined list of infections in the protocol^5^. While baseline data were complete, substantial missingness in HRQoL data was observed in 12-month questionnaires and HRQoL components linked to primary events. Missing utility data were imputed using multiple imputation by kernel nearest neighbour matching, conditioning on treatment allocation, baseline HRQoL, and other covariates mentioned above. Complete first year utilities were estimated by averaging the QALYs scores over imputed datasets for each individual.

#### Costs and QALYs for year 2 onwards

From the beginning of the second and up to the end of the fifth year of analysis, costs and QALYs were assumed to accrue at an annual constant rate to each patient according to the their status at the 12 month ALABAMA trial end-point in terms of allergy label (having a PAL vs no PAL) and having experienced antibiotic treatment failure within the ALABAMA trial (treatment failure vs no treatment failure). Instead of applying fixed costs and QALY values to each year after the end of ALABAMA, we considered the alternative of modelling the annual transitions of the patient cohort between de-labelling and relabelling states, but we could not find reliable information for a UK setting form the literature review that could indicate the likely magnitude of such events, and discussions with clinical experts co-investigators in ALABAMA suggested that the probability of re-labelling or subsequent de-labelling was likely to be small. Therefore, we opted for a simple approach where we investigated how a fixed cumulative annual rate of cost and QALY effects of PAAP would affect its long-term cost-effectiveness.

The annual costs for the years 2 to 5 in the model were estimated from a count regression analysis of quantities of resource use for each category of costs separately. That is, for secondary care, we ran three separate regression models, one each to estimate the number of hospital admissions, outpatient attendances, and attendances to the emergency department; for primary care we ran two separate regression models, one to estimate the number of primary care (GP or nurse-led) consultations and the other to estimate the number of prescription medications. These analyses included all available observations from the second year after baseline for those who were not de-labelled within the 12 months duration of ALABAMA or from the second year after de-labelling for trial participants de-labelled within the trial. The data were split in annual periods and analysed using Poisson regression models with an offset covariate to account for the amount of time at risk trial participants contributed data for the respective year i.e. from 1 to 365 days, and an adjusting covariate set including age, gender, number of QOF conditions, number of antibiotic prescriptions at baseline and the indicators of having had treatment failure during ALABAMA (yes/no) and having a PAL (yes/no) at the 12-month trial end time point. We also included random effects that vary between trial participants but remained fixed across repeated annual observations of the same individual and corresponding random effects for GP practice; due to the long time to compute these regressions, the final analysis excluded the individual participant random effects as this produced only small differences in results.

In order to estimate the costs for each of these quantities of resource use we ran linear regressions of the ratio of total annual costs to the total annual quantities of each of the three secondary care services (inpatient, outpatient and emergency department attendance) and the two primary care resource (consultations and antibiotic prescriptions) categories against the treatment failure and PAL status indicators and the same covariate set as for the regression of quantities of resource use. We obtain marginal predictions for the four conditions defined by the combination of treatment failure and PAL status by aggregating over the covariate set in the sample and adding up the product of predicted price times quantity across the 5 cost categories to derive total costs. It must be noted that by not including a covariate for treatment allocation the implicit assumption was made that resource use and costs were independent of randomised allocation conditional on de-labelling status and treatment failure in all these analyses.

Table A1.3 presents the predicted annual costs pay-offs for the four PAL status-PAL allergy conditions, which were added to the costs and QALYs accrued within the trial to the subset of trial participants at the end of each decision tree branch in Figure 1 according to their combined PAL and treatment failure status at 12 months.

**Table A1.3: Annual costs as a function of PAL status and treatment failure during ALABAMA trial ^a^**

| **Cost Pay-off** | **Point Estimate**  **(N=532 participants, 818 periods)** | **95% CI** | |
| --- | --- | --- | --- |
|  |  | **Lower bound** | **Upper bound** |
| **No PAL at 12 months and no TF** | 796.89 | 581.13 | 1012.65 |
| **No PAL at 12 months and had TF** | 1107.66 | 602.61 | 1612.69 |
| **Retain PAL at 12 months and no TF** | 848.58 | 653.68 | 1043.49 |
| **Retain PAL at 12 months and had TF** | 1239.81 | 815.65 | 1663.97 |

*Notes:* Analysis adjusted for trial baseline minimisation factors (age, number of prescriptions in the 24 months prior, number of QoF conditions and GP practice) and gender, using separate Poisson regressions for each of hospital admissions, outpatient visits, emergency attendances, primary care consultations and number of prescriptions in primary care; analysis also adjusted unit costs (i.e. ratio of total costs to the resource use category count) of each cost category for the same covariates in Ordinary Least Squares regressions. PAL: Penicillin Allergy Label; TF: Treatment Failure.

Unlike resource use data, QALY data were only available within the trial. We therefore assumed that the QALY values accrued by trial participants within the 12-month ALABAMA trial period applied to the first year of the model-based analysis and remained the same for each of the subsequent four years of the analytical time horizon.

The full set of model parameter values for the base case analysis and probability distributions for PSA are presented in Table A1.4. As no individuals were de-labelled following positive test results, their costs and QALYs were set to zero both in the base case analysis and PSA. For other branches with zero individuals included in the trial, we used year 2+ annual costs and average year 1 QALYs by de-labelling and treatment failure status as proxies for the first-year costs and QALYs, respectively.

**Table A1.4: Model parameter values and probability distributions**

|  | **PAAP (N=401)** | | **Usual care (N=410)** | |  |
| --- | --- | --- | --- | --- | --- |
| **Model Parameter** | **Point estimate** | **SD** | **Point estimate** | **SD** | **Distribution** |
| *Event Probabilities* | | | | | |
| 1. Receive test | 0.910 | 0.082 | 0.012 | 0.012 | Beta |
| 2. Test positive | 0.082 | 0.075 | 0.200 | 0.160 | Beta |
| 3. PAL removed after positive test | 0.000 | Fixed | 0.000 | Fixed | Beta |
| 4. PAL removed after negative test | 0.973 | 0.026 | 1.000 | Fixed | Beta |
| 5. TF after test positive & PAL not removed | 0.033 | 0.032 | 0.000 | 0.000 | Beta |
| 6. TF after test positive & PAL removed | 0.000 | 0.000 | 0.000 | 0.000 | Beta |
| 7. TF after test negative & PAL not removed | 0.111 | 0.099 | 0.000 | 0.000 | Beta |
| 8. TF after test negative & PAL removed | 0.089 | 0.081 | 0.000 | 0.000 | Beta |
| 9. TF with no test | 0.083 | 0.076 | 0.114 | 0.101 | Beta |
| *Cost* | | | | | |
| *First year costs* | | | | | |
| 10. Test+, PAL & TF | 510.226 | 0.000 | 0.000 | 0.000 | Lognormal |
| 11. Test+, PAL & No TF | 1176.551 | 1587.103 | 839.634 | 0.000 | Lognormal |
| 12. Test+, No PAL & TF | 0.000 | 0.000 | 0.000 | 0.000 | Lognormal |
| 13.Test+, No PAL & No TF | 0.000 | 0.000 | 0.000 | 0.000 | Lognormal |
| 14. Test-, PAL & TF | 3565.614 | 0.000 | 0.000 | 0.000 | Lognormal |
| 15. Test-, PAL & No TF | 501.371 | 399.853 | 0.000 | 0.000 | Lognormal |
| 16. Test-, No PAL & TF | 1868.200 | 2249.552 | 0.000 | 0.000 | Lognormal |
| 17. Test-, No PAL & No TF | 1481.029 | 2694.723 | 409.135 | 724.907 | Lognormal |
| 18. No Test & TF | 432.468 | 333.952 | 2077.519 | 2760.803 | Lognormal |
| 19. No Test & No TF | 604.887 | 1063.404 | 1208.449 | 3038.706 | Lognormal |
| *Annual costs for years 2 to 5* | | | | | |
| 20. No PAL & no TF | 796.891 | 110.082 | 796.891 | 110.082 | Lognormal |
| 21. No PAL & TF | 1107.655 | 257.679 | 1107.655 | 257.679 | Lognormal |
| 22. PAL & no TF | 848.584 | 99.442 | 848.584 | 99.442 | Lognormal |
| 23. PAL & TF | 1239.812 | 216.412 | 1239.812 | 216.412 | Lognormal |
| *Annual QALYs* | | | | | |
| 24. Test+, PAL & TF | 0.924 | 0.000 | 0.000 | 0.000 | Beta |
| 25. Test+, PAL & No TF | 0.828 | 0.226 | 0.980 | 0.000 | Beta |
| 26. Test+, No PAL & TF | 0.000 | 0.000 | 0.000 | 0.000 | Beta |
| 27.Test+, No PAL & No TF | 0.000 | 0.000 | 0.000 | 0.000 | Beta |
| 28. Test-, PAL & TF | 0.776 | 0.000 | 0.000 | 0.000 | Beta |
| 29. Test-, PAL & No TF | 0.837 | 0.132 | 0.000 | 0.000 | Beta |
| 30. Test-, No PAL & TF | 0.808 | 0.146 | 0.000 | 0.000 | Beta |
| 31. Test-, No PAL & No TF | 0.880 | 0.137 | 0.888 | 0.091 | Beta |
| 32. No Test & TF | 0.689 | 0.060 | 0.755 | 0.249 | Beta |
| 33. No Test & No TF | 0.902 | 0.082 | 0.850 | 0.175 | Beta |
| *Average QALYs by PAL and TF status (only used as proxies)* | | | | | |
| 34. PAL & TF | 0.755 | 0.239 | 0.755 | 0.239 | Beta |
| 35. PAL & No TF | 0.853 | 0.173 | 0.853 | 0.173 | Beta |
| 36. No PAL & TF | 0.808 | 0.146 | 0.808 | 0.146 | Beta |
| 37. No PAL & No TF | 0.880 | 0.136 | 0.880 | 0.136 | Beta |

*Notes:* These values were used to substitute missing QALY when there was no individual for certain decision tree branches. TF: Treatment Failure; SD: Standard Deviation; PAL: Penicillin Allergy Label.

**Supplementary File 2. Probabilistic Sensitivity Analysis Methods**

We assumed that the trial was representative of the situation that we would like to model. To determine the distributions of probability values, we employed a Bayesian analysis and set a uniform prior that is equivalent to a Beta distribution with parameters (1,1), indicating minimal prior information. The data were then assumed to follow a binomial distribution to calculate the posterior distribution. As noted above, no individuals should be de-labelled with positive test results, so the probabilities remained zero. Due to the tiny probabilities of individuals in the usual care arm who experienced treatment failure conditioning on test results and de-labelling status, we assumed these parameters followed the same distributions as the corresponding parameters from the treatment arm. We also specified uncertainty distributions for the costs and QALYs associated with different pathways in the decision tree model. Log-normal distributions were used for costs, as they are positive and heavily skewed due to some individuals experiencing very high costs of care. For QALYs, we used Beta distributions, as quality of life weights typically fall between 0 and 1, similar to probabilities. The PSA involves sampling from the assigned distributions for each parameter. The model was run 5,000 times, with each iteration randomly selecting parameter values from their respective distributions. Mean costs and mean QALYs were then calculated by averaging the results across all 5,000 simulations.

**Supplementary File 3. Value of Information Analysis Methods**

A total sample size between 1592 and 2090 participants provided 80-90% power to detect a clinically important absolute difference of 7.9% in re-prescription rate at one year between groups (i.e. reducing from 19.8% in the control group to 11.9% in the PAAP group) at 5% level of significance (2-sided). The sample size had been adjusted assuming 50% of participants would require at least one prescription within 1 year from randomisation and allowing for 10% dropout. Participants are classed as enrolled at the point of randomisation. Given the achieved sample size by ALABAMA, the new trial (‘ALABAMA2’) would require enrolling 769 and 1267 new patients to achieve the original power in ALABAMA.

The costs of the new hypothetical ALABAMA2 trial at the two sample sizes were estimated using cost information and monthly recruitment rates observed with the existing capacity of 51 GP practices in ALABAMA. The recruitment rate of 30 patients per month observed during the period January 2022 to April 2023, when the distortions from the pandemic on recruitment were no longer affecting the trial, was used. This results in a trial that required 30 months of recruitment to achieve 80% power or 46 months of recruitment to achieve 90% power. Other, fixed costs of ALABAMA2 involved 6 months of trial set-up, 12 months of follow-up and 6 month of analysis, plus £10000 of dissemination costs. Primary and secondary care data linkages would also be required as per ALABAMA, providing longer follow-up data beyond the 12-month trial end-point for patients recruited earlier into the new trial. Excess treatment costs to primary care to cover the provision of screening for eligibility by GPs and to allergy centres for the provision of penicillin allergy testing and antibiotic medications to intervention and usual care arm trial participants are also accounted for in the calculations.

We present the results as the Expected Net Value of Sampling as the difference between the expected value of sample information times the eligible population (i.e. the population EVSI) and the cost of the trial. The population EVSI was calculated as the product of the total adult population of England (45.7m in midyear 2023) times the proportion of the adult English population meeting the trial inclusion criteria (i.e. having at least one antibiotic prescription in the last 24 months). In the absence of a reliable data on the latter parameter, the observed 2.6 percent rate of trial eligibility was applied resulting in 1.195m. Using the observed trial recruitment rate of 6.2% as a conservative rate of conversion, we obtain the estimated 74,325 absolute prevalent number of patients that would be able to benefit from the new testing service to which additional smaller flows of incidental cohorts would be added each year. At the geometric average 0.7% rate of growth in the English adult population over 1999 and 2039 (ONS 2023) and assuming the rate of eligibility and conversion rates remain the same, the eligible population in our analysis is estimated to be 79,877 over the next 10 years.

We implement the EVSI estimation using the regression-based approach ^13^. We use the 10,000 simulations of model inputs and the NMB output from the probabilistic sensitivity analysis of the decision tree model and augment it with the simulation of outcomes that would be observed in ALABAMA2 when recruiting 769 and, alternatively, 1267 patients conditional on the simulated parameter values for events probabilities, costs and utility parameter pay-offs listed in Table A1.4 ^14^.

**Supplementary File 4. Cost-effectiveness plane and Cost-effectiveness Acceptability Curve**

Figure A4.1 illustrates the uncertainty in the cost-effectiveness results, showing the PSA estimates of incremental costs for PAAP on the vertical axis and incremental QALYs on the horizontal axis. The diagonal line represents the decision threshold of £20,000 per QALY. Points above this line indicate a cost per QALY gained higher than the threshold, meaning PAAP is not considered cost-effective, while points below the line indicate a cost per QALY gained below the threshold, suggesting cost-effectiveness. The proportion of points below the diagonal line reflects the probability that PAAP is cost-effective, which is 48% as shown in Table 3. The mean ICER value calculated across 5,000 simulations falls slightly below the decision threshold line, suggesting cost-effectiveness, while the median ICER is above the threshold, indicating otherwise. Despite the mean ICER suggesting PAAP is cost-effective, the probability of it being cost-effective remains below 50%.

**Figure A4.1: Cost-effectiveness plane with mean and median ICERs^a^**


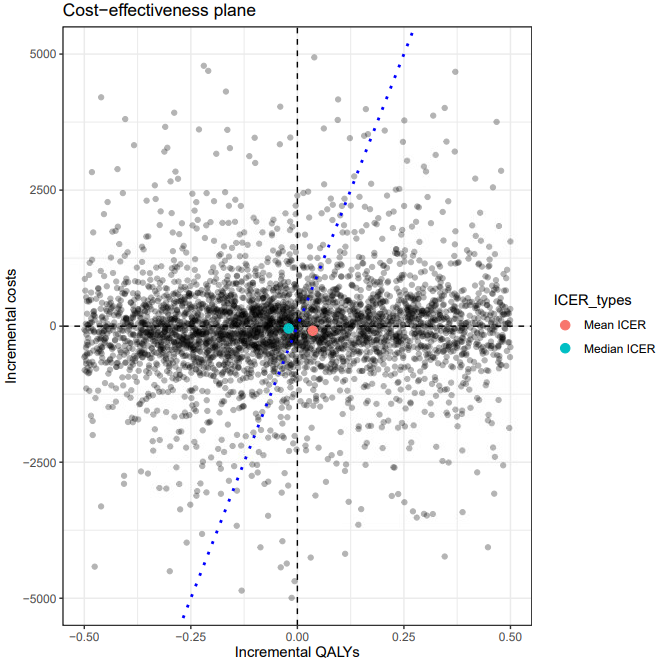


^a^ ICER: incremental cost-effectiveness ratio (i.e. incremental cost per QALY gained by PAAP)

Figures A4.2 and A4.3 illustrate the probability of cost-effectiveness as a function of willingness to pay per QALY for year 1 and the 5-year period, respectively. Figure A4.2 shows an upward-sloping CEAC curve, indicating that the probability of PAAP being cost-effective increases as willingness to pay rises from £0 to £10,000. In contrast, Figure A4.3 reveals a decline in cost-effectiveness probability, as most simulations suggest PAAP generates fewer QALYs than usual care (see median ICER in Figure A4.1), before stabilizing at 48%. Even at higher willingness-to-pay thresholds, substantial uncertainty remains, highlighting the potential value of further research to refine these estimates, as explored in the subsequent VOI analysis.

**Figure A4.2: Cost Effectiveness Acceptability Curve in year 1**


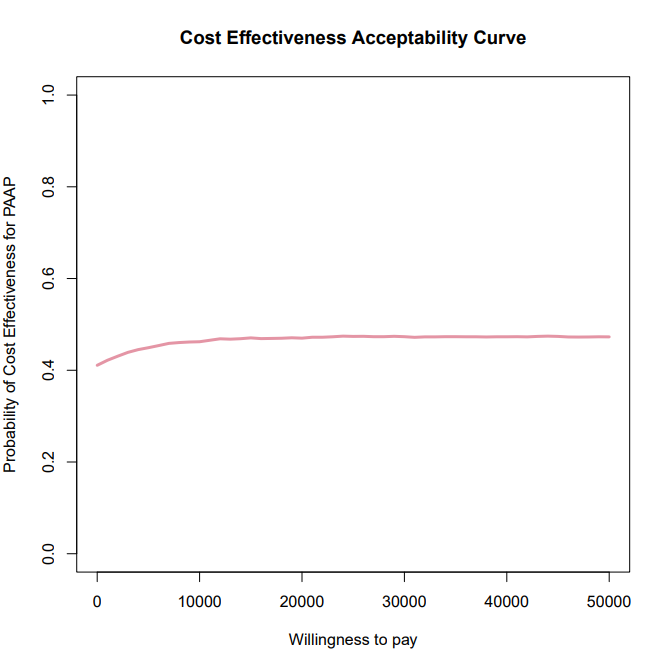


**Figure A4.3: Cost Effectiveness Acceptability Curve over 5-year period**
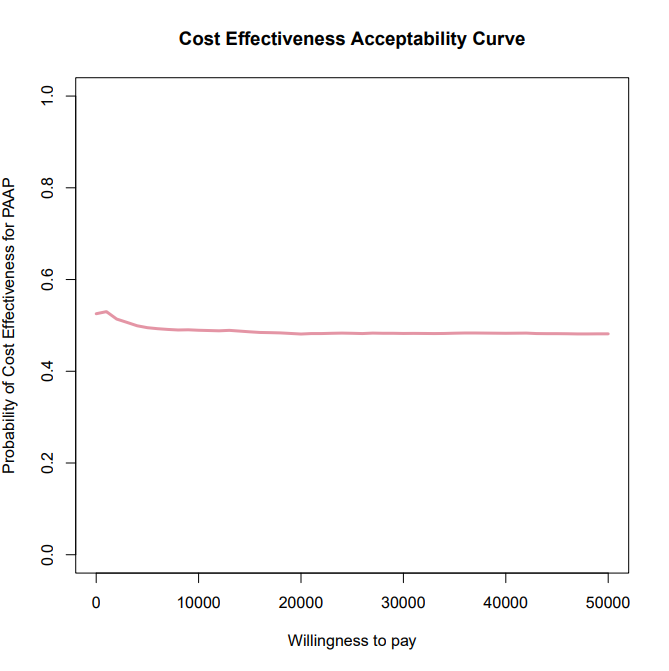

Supplement: online supplemental file 1 [file bmjopen-15-12-s001.docx]
