# Supplementary material for: The cost-effectiveness of penicillin allergy assessment pathway (PAAP): a decision analysis
Source: BMJ Open. 2025 Dec 3;15(12):e104168. doi: 10.1136/bmjopen-2025-104168 (PMC12682163; doi:10.1136/bmjopen-2025-104168)
Supplement: Supplementary file 2 [file bmjopen-2025-104168.reviewer_comments.pdf]

PEER REVIEW HISTORY

BMJ Open publishes all reviews undertaken for accepted manuscripts. Reviewers are asked to complete a checklist review form and are provided with free text boxes to elaborate on their assessment. These free text comments are reproduced below.

ARTICLE DETAILS

Title (Provisional)

The cost-effectiveness of penicillin allergy assessment pathway (PAAP): a decision analysis

Authors

Yang, Miaoqing; Bestwick, Rebecca; Howdon, Daniel; Ahmed, S.; Powell, Neil; Armitage, Kelsey Fiona; Fielding, Joanne; Porter, Catherine; Savic, Sinisa; West, Robert M; Howard, Philip; Galal, Ushma; Pavitt, Sue; Sandoe, Jonathan; Mujica-Mota, Ruben

---

VERSION 1 - REVIEW

**Reviewer**	**1**
**Name**	**Chow, Timothy**
**Affiliation**	**UT Southwestern Medical Center**
**Date**	**03-Jun-2025**
**COI**	**None**

The authors present a secondary analysis of the ALABAMA RCT of a penicillin allergy assessment pathway, extrapolating the first 12 months of data over 5 years to assess cost-effectiveness.

The authors address an important question as our field considers best practices in implementing PAL delabeling equitably in an environment of finite resources, where cost-effectiveness is an important metric for key stakeholders.

The article is overall well-written but in my opinion, there are a few points that need to be addressed to clarify the scope and context of their analyses as outlined below:

Major Comments:

Lines 139-140: The authors state that the ALABAMA trial was terminated early due to COVID-19. It seems like extrapolating data from a 12 month period of healthcare utilization that fell in the midst of an emerging pandemic may not be directly generalizable to subsequent year healthcare utilization. The authors need to expand in the discussion around lines 311-313 to discuss further this limitation and how they accounted for this.

Lines 147: If this study is an extrapolation of the 12 month ALABAMA results, please state why 5 years was determined as a cutoff.

Lines 151-152: The presentation of the factors included for analysis would be strengthened by a graphical representation particularly since many details are presented in the supplementary materials, see Figure 2 from the following article for an excellent example: Sousa-Pinto B, Blumenthal KG, Macy E, Pereira AM, Azevedo LF, Delgado L, Fonseca JA. Penicillin Allergy Testing Is Cost-Saving: An Economic Evaluation Study. Clin Infect Dis. 2021 Mar 15;72(6):924-938. doi: 10.1093/cid/ciaa194. PMID: 32107530; PMCID: PMC7958749.

Lines 260-267: The ALABAMA trial had the primary outcome in terms of looking at "treatment failure defined as representation with worsening or non-resolving or new symptoms following treatment with an antibiotic up to 28 days after initial antibiotic prescription". This is an important outcome but not the only negative health outcome that has been associated with penicillin allergy labels; when we are thinking about an economic evaluation of a PAL delabeling intervention, I am interested in the broad economic implications. Can the authors comment in the discussion about how the analysis is impacted by this choice of primary outcome and whether or not it would change if the outcome as different (for example if the primary outcome was a persistent penicillin allergy label). It's possible that this analysis does capture other outcomes that weren't the primary outcome in the original trial, but it's not clear to me that this is the case and why.

Minor Comments:

Figure 1: typo, "treatment failture" numerous times

Line 279: There is a difference between health benefits and cost-effectiveness. PAL delabeling has significant health benefits across age groups, but may have differing cost-effectiveness across different demographics based on other factors. I would suggest rewording this.

**Reviewer**	**2**
**Name**	**Rose , Morgan**
**Affiliation**	**Austin Health, Department of Infectious Diseases**
**Date**	**15-Jun-2025**
**COI**	**None**

I congratulate the authors on addressing this important question within the field of antibiotic allergy. One of the current challenges in penicillin allergy is how we expand existing allergy assessment services to address the burden of allergy labels in the community. Primary care offers a unique setting to deliver accessible low-risk allergy testing

and delabeling. Exploring the cost-effectiveness of a shared primary care/specialist assessment model is an important step in developing long-term pathways of community allergy testing.

Overall I feel that the manuscript was well designed and undertaken. However, a few minor revisions could be considered to better contextualise this manuscript within more recent research:

Discussion: I noted the relatively more strict exclusion criteria of the ALABAMA study (e.g. excluding pts receiving beta-blocker therapy). A broader inclusion for direct oral challenge eligibility (such as that seen in the PALACE study (doi: 10.1001/jamainternmed.2023.2986.)) could direct more patients to cheaper GP-based delabeling and may further increase the cost-effectiveness of primary care allergy testing models. Discussion of the impact of slightly more inclusive newer assessment models (such as PEN-FAST used in the PALACE study) would be of value in informing future explorations of allergy testing in primary care.

Lines 273-276: I would appreciate more data regarding the patients in the PAAP arm that were not tested. In particular more detailed demographic, comorbidities data and reasons for not proceeding with testing if available.

Lines 277-281: Highlighting subgroups in whom delabeling was most cost effective mirrors findings from inpatient allergy assessment studies (Chua et al doi: 10.1093/cid/ciaa653, and Hannah et al https://doi.org/10.1111/imj.16543). Whilst unrestricted programs can functional effectively, this data also supports the concept of targeted testing programs in settings with fewer resources for allergy assessment.

Line 307: The authors could consider including more recent relabelling data including that from the PALACE study (6 month follow-up) doi: 10.1001/jamanetworkopen.2024.29621

Lines 311-316: The inclusion of a cost benefit analysis of future studies and their contribution to cost-effectiveness data is an important addition to this manuscript and contributes meaningfully in support of future funding for non-allergist assessment programs.

**Reviewer**	**3**
**Name**	**Pasin, Ozge**
**Affiliation**	**University of Health Sciences, Istanbul**
**Date**	**09-Jul-2025**
**COI**	**None**

The authors report several key variables (such as costs and healthcare utilization) using means and standard deviations despite visibly high standard deviations suggesting potential non-normality and skewed distributions. It would be more appropriate to present medians

and interquartile ranges (Q1–Q3) for these variables to better reflect the central tendency and dispersion, particularly for cost data, which is known to be highly skewed in health economic evaluations.The manuscript would benefit from including a dedicated ‘Statistical Analysis’ section where all analytical methods used (including regression modeling, handling of missing data, sensitivity analyses, subgroup analyses, and Value of Information calculations) are systematically described. This would improve the transparency, reproducibility, and interpretability of the results, particularly for readers who are less familiar with advanced health economic modeling.The manuscript mentions the use of R software for decision tree modeling but does not provide sufficient detail about the statistical software and specific packages used for the various analyses, including sensitivity analyses, imputation, and Value of Information calculation. Including such details (software name, version, key packages) would enhance reproducibility and transparency.

**Reviewer**	**4**
**Name**	**Dack, Kyle**
**Affiliation**	**University of Bristol, MRC Integrative Epidemiology Unit**
**Date**	**26-Jul-2025**
**COI**	**None**

Statistical review summary:

The methodology is clearly described, and the statistical analysis is sound. The results are reported reasonably and the interpretation includes a fair discussion of the limitations. My primary suggestion is that currently the use of different data sources is slightly unclear, and clarifying this would improve the methodology section.

Abstract:

1. The study involves a decision tree analysis based on data from a prior RCT. However, lines 72-74 describe a study setting and participant information in a manner which may confuse readers or make them think this was a new trial. Since the analysis involves a theoretical model and secondary data from a prior RCT, I suggest revising these lines to clarify, and/or condensing to a “data source” subheading to make it clear there is no setting/participants in the typical sense (this is also consistent with the methods section of the paper which does not use these headings).

2. Line 81: are the units for 801 and 1,167 in GBP?

Introduction:

3. Clear and well supported. No suggestions.

Methods:

4. Readers may be curious why 5 years was chosen as the time horizon. Line 93 commented that this was relatively short – what informed the decision not to attempt a longer horizon? I suggest this could be briefly described in the methods.

5. Lines 151-159. I suggest placing these under an “outcomes” subheading, as it currently feels a little displaced.

6. Line 149: I suggest adding a brief high-level summary of what data was taken from the RCT, and what from the systematic review.

7. Line 149: Is local health costing data not a third component of the analysis? This is mentioned in Line 24 of the supplementary file – data is taken from local hospitals/clinics related to staff and other costs. It sounds as if this is independent from the RCT data?

8. There are references to a systematic review on lines 70 and 149, which gave the impression this would be a core part of the methodology / data sources. But the remainder of the paper does not mention a systematic review at all, and I am unsure which systematic review this was and what data was taken from it. I suggest clarifying which model inputs were informed by it, or whether it was used to test certain assumptions.

9. Line 162: suggest clarifying “penicillin allergy testing” on the first reference to it in each section, to help readers understand that subsequent mentions of “testing” are shorthand for it.

10. The statistical analysis is described with a good level of detail while remaining understandable to a non-statistical audience. The selected methods are all reasonable. My primary suggestion regarding the methods is to clarify the various data sources, which specific data was taken from each, and how it was used. Potentially a separate “data sources” subheading could clarify this, including the RCT, systematic review, local health data, and any other sources. It may add a slight amount of repetition but would likely be a help to readers.

Results

11. Everything is reported clearly and reasonably in-text.

12. Table 1. Suggest adding a footnote defining SPT, STOC, DPC, and the units of the costs section, as is presented in other tables.

13. Figure 2. Is it possible to replace the variable names with more reader friendly labels? This would be possible in R by changing the column names themselves, which could include spaces, and then referenced in the code using backticks.

VERSION 1 - AUTHOR RESPONSE

Reviewer: 1
Dr. Timothy Chow, UT Southwestern Medical Center
Comments to the Author:
The authors present a secondary analysis of the ALABAMA RCT of a penicillin allergy assessment pathway, extrapolating the first 12 months of data over 5 years to assess cost-effectiveness.
The authors address an important question as our field considers best practices in implementing PAL delabeling equitably in an environment of finite resources, where cost-effectiveness is an important metric for key stakeholders.

The article is overall well-written but in my opinion, there are a few points that need to be addressed to clarify the scope and context of their analyses as outlined below:

Major Comments:
Lines 139-140: The authors state that the ALABAMA trial was terminated early due to COVID-19. It seems like extrapolating data from a 12 month period of healthcare utilization that fell in the midst of an emerging pandemic may not be directly generalizable to subsequent year healthcare utilization. The authors need to expand in the discussion around lines 311-313 to discuss further this limitation and how they accounted for this. *Thanks for your suggestion. We've added relevant information now in the discussion section.*
Lines 147: If this study is an extrapolation of the 12 month ALABAMA results, please state why 5 years was determined as a cutoff. *Thank you. We chose 5 years because according to our systematic review of the economic literature (currently under review for publication) that is the longest follow-up period for which data are available on healthcare use associated with penicillin allergy labels (Sousa-Pinto et al. 2021). We have added this reason to this line and a comparison of ours with Sousa et al findings in the Discussion.*
Lines 151-152: The presentation of the factors included for analysis would be strengthened by a graphical representation particularly since many details are presented in the supplementary materials, see Figure 2 from the following article for an excellent example: Sousa-Pinto B, Blumenthal KG, Macy E, Pereira AM, Azevedo LF, Delgado L, Fonseca JA. Penicillin Allergy Testing Is Cost-Saving: An Economic Evaluation Study. Clin Infect Dis. 2021 Mar 15;72(6):924-938. doi: 10.1093/cid/ciaa194. PMID: 32107530; PMCID: PMC7958749. *Thanks for your suggestion. A similar figure has been added.*

Lines 260-267: The ALABAMA trial had the primary outcome in terms of looking at "treatment failure defined as representation with worsening or non-resolving or new symptoms following treatment with an antibiotic up to 28 days after initial antibiotic prescription". This is an important outcome but not the only negative health outcome

that has been associated with penicillin allergy labels; when we are thinking about an economic evaluation of a PAL delabeling intervention, I am interested in the broad economic implications. Can the authors comment in the discussion about how the analysis is impacted by this choice of primary outcome and whether or not it would change if the outcome as different (for example if the primary outcome was a persistent penicillin allergy label). It's possible that this analysis does capture other outcomes that weren't the primary outcome in the original trial, but it's not clear to me that this is the case and why. *Thank you for the comment. We agree that treatment failure is only one of several important outcomes associated with penicillin allergy labels. In our published within-trial analysis (Sandoe et al. 2025), we examined other outcomes, including penicillin prescribing, total antibiotic use, hospital admissions, and delabelling rates (see 'Outcomes' section and Table 2 of the publication). These outcomes were incorporated into the cost and QALY inputs in the economic evaluation to reflect the wider implications of penicillin allergy labels and their removal. We have added a sentence to clarify this at the end of these lines. Please note in the existing Discussion that, in addition to treatment failure, penicillin allergy label at 12 months was the driver of our results (lines 338-344 in new numbering) and we also explored the impact of relabelling rates in subsequent years in a scenario analysis and found similar results (lines 349-352 in new numbering). We have updated results and Discussion of this scenario analysis to reflect recent evidence on relabelling rates suggested by Reviewer 2 below. Please let us know if you would think more clarification would be desirable.*

Minor Comments:

Figure 1: typo, "treatment failture" numerous times *Thanks. The typo has been corrected.*

Line 279: There is a difference between health benefits and cost-effectiveness. PAL delabeling has significant health benefits across age groups, but may have differing cost-effectiveness across different demographics based on other factors. I would suggest rewording this. *We have added the following underlined words for clarity: "These results suggest targeting the PAAP model to, say, all males over 65 and females older than 40 who have used antibiotics in the past 12-24 months may yield the greatest per-patient net health benefit at a population level with existing health system resources and relieve pressure on overstretched allergy testing services". Please let us know if we have missed any points from your comment. Thank you.*

Reviewer: 2

Dr. Morgan Rose , Austin Health, Peter MacCallum Cancer Centre

Comments to the Author:

I congratulate the authors on addressing this important question within the field of antibiotic allergy. One of the current challenges in penicillin allergy is how we expand existing allergy assessment services to address the burden of allergy labels in the

community. Primary care offers a unique setting to deliver accessible low-risk allergy testing and delabeling. Exploring the cost-effectiveness of a shared primary care/specialist assessment model is an important step in developing long-term pathways of community allergy testing.

Overall I feel that the manuscript was well designed and undertaken. However, a few minor revisions could be considered to better contextualise this manuscript within more recent research:

Discussion: I noted the relatively more strict exclusion criteria of the ALABAMA study (e.g. excluding pts receiving beta-blocker therapy). A broader inclusion for direct oral challenge eligibility (such as that seen in the PALACE study (doi: 10.1001/jamainternmed.2023.2986.)) could direct more patients to cheaper GP-based delabeling and may further increase the cost-effectiveness of primary care allergy testing models. Discussion of the impact of slightly more inclusive newer assessment models (such as PEN-FAST used in the PALACE study) would be of value in informing future explorations of allergy testing in primary care.

The ALABAMA trial had more strict exclusion criteria than some observational studies, but beta blocker use is also an exclusion in a number of these studies (Powell et al. 2023). The inclusion and exclusion criteria for the ALABAMA and PALACE trials were very similar (please see Table 1). In the trial flow diagram data are presented which show that the ALABAMA study only excluded patients receiving beta-blocker therapy if they could not delay taking their beta-blocker until after testing (Armitage et al. 2023), which only accounted for 2 exclusions in the trial (Sandoe et al. 2025). We therefore disagree that the trial had markedly more strict exclusion criteria. The PALACE trial is an excellent piece of work and the findings support undertaking direct oral challenge testing in low risk patients, rather than skin testing, which is what was done in ALABAMA.

GP/primary delivered may well be a more cost-effective approach to penicillin allergy delabelling, but this has not been assessed adequately in the literature. Please note that the ALABAMA trial identified participants from general practice lists and involved primary care clinicians consenting and referring patients, but all participants were referred to secondary care for assessment and testing, thus ALABAMA was not a primary care delivered delabelling initiative. There are several barriers to GPs delabelling patients in primary care, perhaps most notably, a reluctance to take responsibility for removing a label based on clinical assessment alone (Wanat et al. 2019, Wanat et al. 2021).

PEN-FAST may provide a useful way to risk stratify patients in terms of risk of a true allergy but does not remove the need to assess a patient's suitability for testing, so questions about medications and past medical history are still required. In addition, patients with anaphylaxis have been misclassified using this tool, the risk of adverse reactions arising from misclassification would need to be added into the assessment

(Ghiordanescu et al. 2024). We are not aware that PEN-FAST speeds up the process of assessment compared to other risk stratification tools (it is quicker than using skin testing), which would be the only basis to propose that PEN-FAST provides a more cost-effective approach (Copaescu et al. 2023).

We have added the following text to the discussion:

"Other service delivery models, including through the use of decision support tools, such as "PEN-FAST" in the PALACE trial[7] may impact on cost effectiveness, but this would need prospective study. Primary care delivered delabeling may further increase the cost-effectiveness by avoiding the need to travel to hospital but this would also need prospective evaluation."

Table 1: Comparing eligibility criteria in PALACE and ALABAMA trials.

PALACE	ALAMABA
**Inclusion**:	
1. Adult patients (≥ 18 years) referred to the outpatient allergy clinic for a penicillin allergy history;	Male or female, aged 18<br><br>Current penicillin allergy (or sensitivity) record of any<br>kind in their electronic health record
2. Willing and able to give consent	Patient is willing and able to give informed consent for participation in the trial<br>Prescribed systemic antibiotics in the previous 24 months (NB this was because antibiotic prescribing was the primary outcome and the population needed to be enriched with higher antibiotic users)
**Exclusion:**	
1. Patient age is < 18 years;	
2. Patients with a PEN-FAST score ≥3	
3. Pregnancy	Pregnant or Breastfeeding mothers
4. Any other illness that, in the investigator's judgement, will substantially increase the risk associated with the subject's participation in this study, including neurological or psychological conditions;	Considered unsuitable for trial participation by the GP for example, because of chaotic lifestyle,<br>History of brittle/severe asthma or has had a course of steroids in the past 3 months for asthma or unstable coronary artery disease, or severe/poorly controlled skin conditions (GPs may also want to exclude vulnerable patients who are deemed to be unsuitable to participate for other reasons such as, but not limited to, terminal illness, reliability, mental illness, learning difficulties, anxiety, other family circumstances
5. Patients with a history of type A adverse drug reaction, drug-associated anaphylaxis, idiopathic	Allergy history consistent with anaphylaxis to penicillin<br>– History of toxic epidermal necrolysis, Stevens-Johnson syndrome, Drug reaction with

urticaria/anaphylaxis, mastocytosis, serum sickness, blistering skin eruption or acute interstitial nephritis;	eosinophilia and systemic symptoms (DRESS) or any severe rash which blistered or needed hospital treatment, and acute generalised exanthematous pustulosis precipitated by a penicillin
6. Patients where the allergy history was not able to be confirmed with the patient;	
7. Patients on concurrent antihistamine therapy	Currently taking antihistamines and unable to temporarily withhold these for 72 hours pretesting
8. Patients receiving more than stress dose steroids (i.e. > 50mg QID hydrocortisone [or steroid equivalent]).	Currently taking (or recently taken) systemic steroids and unable to stop these for 10 days pretesting
	Life expectancy estimated <1 year by GP
	Unable to attend immunology clinic
	Currently taking beta blocker medication, and unable to temporarily withhold these on the day of penicillin allergy testing

Lines 273-276: I would appreciate more data regarding the patients in the PAAP arm that were not tested. In particular more detailed demographic, comorbidities data and reasons for not proceeding with testing if available. *Thank you for your suggestion, we have added the following text to line 294 in the new numbering: "The group of 36 patients allocated to PAAP who did not receive testing were younger (mean age 54 vs. 51 years) but similar in terms of gender (72 vs 71% female) and total number of courses of antibiotics in the 24 months before baseline (mean 1.92 vs 1.91) than the ITT PAAP group;   4 (11%) did not want to undergo testing, one attended the outpatient appointment for testing but could not go ahead due to eczema/dermatitis, 17 did not attend the appointment (47%), and the rest (n=14, 39%) had no reason recorded)."*

Lines 277-281: Highlighting subgroups in whom delabeling was most cost effective mirrors findings from inpatient allergy assessment studies (Chua et al doi: 10.1093/cid/ciaa653, and Hannah et al https://doi.org/10.1111/imj.16543). Whilst unrestricted programs can functional effectively, this data also supports the concept of targeted testing programs in settings with fewer resources for allergy assessment. *Thanks for your suggestion. The reference has been added in the discussion.*

Line 307: The authors could consider including more recent relabelling data including that from the PALACE study (6 month follow-up) doi: 10.1001/jamanetworkopen.2024.29621
Thank you for your suggestion. We have produced a scenario analysis where penicillin allergy relabelling occurs at the rate observed in PALACE and have added text to describe it in Methods section (Line 257 in new numbering) "In addition we explored a

pessimistic scenario where the 6.6% 6-month relabelling rate observed in the PALACE study remained constant over the 5 year follow up in the Markov chain model."

We have also added text in the Results section (Line 260 in the new numbering): "When the Markov chain model is revised to allow for a 6.6% 6-month relabelling rate that remains constant over 5 years, the (deterministic) ICER is equals £1122 per QALY gained."

Lines 311-316: The inclusion of a cost benefit analysis of future studies and their contribution to cost-effectiveness data is an important addition to this manuscript and contributes meaningfully in support of future funding for non-allergist assessment programs. *Thanks for your comment.*

Reviewer: 3
Dr. Ozge Pasin, University of Health Sciences, Istanbul, Bezmialem Vakif University
Comments to the Author:
The authors report several key variables (such as costs and healthcare utilization) using means and standard deviations despite visibly high standard deviations suggesting potential non-normality and skewed distributions. It would be more appropriate to present medians and interquartile ranges (Q1–Q3) for these variables to better reflect the central tendency and dispersion, particularly for cost data, which is known to be highly skewed in health economic evaluations. *Thank you for the comment. We agree that cost data are often skewed in health economic evaluations. However, the cost and QALY input values reported in Table 2 reflect the parameters used in the decision-analytic model, which requires mean values and SD for probabilistic sensitivity analysis. As such, we reported means and standard deviations in line with standard practice in health economics. This reporting format is consistent with the Consolidated Health Economic Evaluation Reporting Standards (CHEERS) guidelines.*

The manuscript would benefit from including a dedicated 'Statistical Analysis' section where all analytical methods used (including regression modeling, handling of missing data, sensitivity analyses, subgroup analyses, and Value of Information calculations) are systematically described. This would improve the transparency, reproducibility, and interpretability of the results, particularly for readers who are less familiar with advanced health economic modelling. *Thank you for the suggestion. The statistical analysis approach, including regression modelling, handling of missing data, and subgroup analyses, has been previously published as part of our within-trial analysis (Sandoe et al. 2025). We have now cited this publication in the main text to improve clarity and transparency. The methods specific to the economic model, including sensitivity analyses and Value of Information calculations, are described in detail in the current manuscript.*

The manuscript mentions the use of R software for decision tree modeling but does not provide sufficient detail about the statistical software and specific packages used for the various analyses, including sensitivity analyses, imputation, and Value of Information calculation. Including such details (software name, version, key packages) would enhance reproducibility and transparency. *Thank you for the comment. We used R (version 4.5.1) for all analyses. The decision tree model was built from scratch using base R functions and general-purpose packages (e.g., dplyr, ggplot2) rather than a dedicated health economic modelling package. Our modelling approach follows the principles of the DARTH framework for decision-analytic modelling in R (https://github.com/DARTH-git). We have now added this information to the Methods section to improve transparency and reproducibility.*

Reviewer: 4
Dr. Kyle Dack, University of Bristol
Comments to the Author:
Statistical review summary:
The methodology is clearly described, and the statistical analysis is sound. The results are reported reasonably and the interpretation includes a fair discussion of the limitations. My primary suggestion is that currently the use of different data sources is slightly unclear, and clarifying this would improve the methodology section.

Abstract:
1. The study involves a decision tree analysis based on data from a prior RCT. However, lines 72-74 describe a study setting and participant information in a manner which may confuse readers or make them think this was a new trial. Since the analysis involves a theoretical model and secondary data from a prior RCT, I suggest revising these lines to clarify, and/or condensing to a "data source" subheading to make it clear there is no setting/participants in the typical sense (this is also consistent with the methods section of the paper which does not use these headings). *Thanks for the suggestion. We have revised the abstract now.*

2. Line 81: are the units for 801 and 1,167 in GBP? *Added the £ signs.*

Introduction:
3. Clear and well supported. No suggestions. *Thanks.*

Methods:
4. Readers may be curious why 5 years was chosen as the time horizon. Line 93 commented that this was relatively short – what informed the decision not to attempt a longer horizon? I suggest this could be briefly described in the methods. *Thank you. We chose 5 years because, according to our systematic review of the economic literature (currently under review for publication), that is the longest follow-up period for which data are available on healthcare use associated with penicillin allergy labels (Sousa-Pinto et al. 2021). We have added this reason to the first paragraph of the Methods*

section and a comparison of ours with Sousa et al findings in the Discussion.

5. Lines 151-159. I suggest placing these under an "outcomes" subheading, as it currently feels a little displaced. *Thanks for the suggestion. The subheading has been added now.*

6. Line 149: I suggest adding a brief high-level summary of what data was taken from the RCT, and what from the systematic review. *Thanks for the suggestion. The summary has been added.*

7. Line 149: Is local health costing data not a third component of the analysis? This is mentioned in Line 24 of the supplementary file – data is taken from local hospitals/clinics related to staff and other costs. It sounds as if this is independent from the RCT data? *Indeed. Relevant information has now been added in the methods section.*

8. There are references to a systematic review on lines 70 and 149, which gave the impression this would be a core part of the methodology / data sources. But the remainder of the paper does not mention a systematic review at all, and I am unsure which systematic review this was and what data was taken from it. I suggest clarifying which model inputs were informed by it, or whether it was used to test certain assumptions. *Thank you for your suggestion. We have revised Line 149 to read: "We developed an economic model to assess the cost-effectiveness of implementing PAAP initiated in primary care from the English NHS perspective over a 5-year time horizon, the maximum number of years for which we had at least partial data on any trial participant (4.3 years) and found resource use data in our systematic review of economic studies of penicillin allergy de-labelling (currently under review for publication). The modelled population included adults with a recorded penicillin allergy (Armitage et al. 2023). A health economic analysis plan was developed and is available upon request. The model structure was informed by our systematic review of economic studies of Penicillin Allergy de-labelling, with model inputs derived from the ALABAMA trial"*

9. Line 162: suggest clarifying "penicillin allergy testing" on the first reference to it in each section, to help readers understand that subsequent mentions of "testing" are shorthand for it. *Thanks for the suggestion. We've revised the terms accordingly.*

10. The statistical analysis is described with a good level of detail while remaining understandable to a non-statistical audience. The selected methods are all reasonable. My primary suggestion regarding the methods is to clarify the various data sources, which specific data was taken from each, and how it was used. Potentially a separate

“data sources” subheading could clarify this, including the RCT, systematic review, local health data, and any other sources. It may add a slight amount of repetition but would likely be a help to readers. *Thanks for the suggestions. Information about data sources have been added in the methods section now.*

Results
11. Everything is reported clearly and reasonably in-text. *Thanks for your comment.*

12. Table 1. Suggest adding a footnote defining SPT, STOC, DPC, and the units of the costs section, as is presented in other tables. *Thank you for noting this omission. We have added the following definitions to the footnotes of Table 1: “SPT: Skin Prick test. STOC: Skin prick test with oral penicillin challenge. DPC: Direct oral penicillin challenge.”*

13. Figure 2. Is it possible to replace the variable names with more reader friendly labels? This would be possible in R by changing the column names themselves, which could include spaces, and then referenced in the code using backticks. *Thank you for flagging this up. We have revised the labels to spell out the words and delete the underscores. Please let us know if you think there is anything else we can do to make the Figure easier to read.*

VERSION 2 - REVIEW

**Reviewer**	**1**
**Name**	**Chow, Timothy**
**Affiliation**	**UT Southwestern Medical Center**
**Date**	**07-Oct-2025**
**COI**	

The authors have adequately addressed all of my comments.
